# Developmental and Nutritional Dynamics of Malpighian Tubule Autofluorescence in the Asian Tiger Mosquito *Aedes albopictus*

**DOI:** 10.3390/ijms25010245

**Published:** 2023-12-23

**Authors:** Anna Cleta Croce, Anna Garbelli, Andrea Moyano, Sara Soldano, Carlos Tejeda-Guzmán, Fanis Missirlis, Francesca Scolari

**Affiliations:** 1 Institute of Molecular Genetics IGM CNR “Luigi Luca Cavalli-Sforza”, Via Abbiategrasso 207, 27100 Pavia, Italy; anna.garbelli@igm.cnr.it (A.G.); andrea.moyano@igm.cnr.it (A.M.); sara.soldano01@universitadipavia.it (S.S.); 2 Department of Biology and Biotechnology, University of Pavia, Via Ferrata 9, 27100 Pavia, Italy; 3 Department of Physiology, Biophysics and Neuroscience, Cinvestav, Mexico City 07360, Mexico; carlos.tejeda@cinvestav.mx (C.T.-G.); fanis@fisio.cinvestav.mx (F.M.)

**Keywords:** mosquito developmental stages, fluorescent granules, dietary intake, tryptophan, principal cells, confocal microscopy, spectrofluorimetric analysis, emission spectra

## Abstract

Malpighian tubules (MTs) are arthropod excretory organs crucial for the osmoregulation, detoxification and excretion of xenobiotics and metabolic wastes, which include tryptophan degradation products along the kynurenine (KYN) pathway. Specifically, the toxic intermediate 3-hydroxy kynurenine (3-HK) is metabolized through transamination to xanthurenic acid or in the synthesis of ommochrome pigments. Early investigations in *Drosophila* larval fat bodies revealed an intracellular autofluorescence (AF) that depended on tryptophan administration. Subsequent observations documented AF changes in the MTs of *Drosophila* eye-color mutants genetically affecting the conversion of tryptophan to KYN or 3-HK and the intracellular availability of zinc ions. In the present study, the AF properties of the MTs in the Asian tiger mosquito, *Aedes albopictus,* were characterized in different stages of the insect’s life cycle, tryptophan-administered larvae and blood-fed adult females. Confocal imaging and microspectroscopy showed AF changes in the distribution of intracellular, brilliant granules and in the emission spectral shape and amplitude between the proximal and distal segments of MTs across the different samples. The findings suggest AF can serve as a promising marker for investigating the functional status of MTs in response to metabolic alterations, contributing to the use of MTs as a potential research model in biomedicine.

## 1. Introduction

Malpighian tubules (MTs) are typical arthropod organs, specialized in the osmoregulation and excretion of protein-derived and xenobiotic wastes [1,2]. The MTs are composed of only two types of cells that collaborate in ion exchange regulation: principal cells primarily regulating potassium fluxes and stellate cells specialized in chloride transport to maintain the electrolyte and water balance [3]. Maintaining a simple histological organization, principal and stellate cells further differentiate along the segments of the MTs [4], while different insect species vary significantly in the number, shape and specialized MT functions that encompass a wide range of roles, including bioluminescence, the processing of proteinaceous products and inorganic salts for adhesive or protective purposes and participation in the immune response [1,5]. The metabolic pathways governing the functions of the MTs have sparked interest, whereas understanding the paracellular and transcellular mechanisms that regulate electrolytes and fluid secretion facilitates comparative ecological and evolutionary studies [6,7]. Besides enhancing fundamental knowledge, interest in MTs is devoted to exploring potential technological applications and their potential roles as valuable models for biomedical research due to shared genetic and molecular pathways between insects and mammals, including humans. The advantages lie in the easy and cost-effective insect rearing, the availability of suitable genetic mutants or transgenic strains and the exemption of experiments involving insects from ethical standards governing the use of animals for scientific purposes [8,9,10]. One such example is the study of *Drosophila melanogaster* (Diptera: Drosophilidae) MTs as a model for investigating the etiology of kidney crystal formation, exploring possible pharmacological treatments for stone disease [9,10,11,12,13,14,15].

Hematophagy is a characteristic trait of female mosquitoes belonging to most genera [16]. This feeding habit has a great impact on the acquisition and transmission of pathogens and makes mosquitoes important vectors of arboviruses of public health importance [17,18,19]. Mosquitoes possess five MTs of comparable functionality with proximal and distal segments that are morphologically distinct. The distal MTs are mainly involved in the excretion of water, electrolytes, wastes and xenobiotics, while the proximal segments reabsorb water and useful electrolytes [20,21]. The paracellular and transcellular mechanisms operating within the MTs to control electrolyte and fluid secretion have been identified as potential targets for the development of innovative vector control strategies [22,23]. Targeting the osmoregulatory mechanisms of the MTs has also been proposed as a strategy to influence metabolism, metabolic waste excretion and antioxidant activity disrupting the life cycle of disease vectors and hindering oviposition [21,24,25].

In this context, the metabolism of the amino acid tryptophan has attracted attention because, beyond its role in protein synthesis, it serves as a precursor to a variety of bioactive molecules, including serotonin, melatonin and kynurenine (KYN) pathway metabolites, acting as hormones and neuronal and immune regulators [26]. The oxidation of tryptophan to KYN is the first step of a catabolic pathway that produces kynurenic acid through transamination or 3-hydroxy kynurenine (3-HK) through hydroxylation, while xanthurenic acid (XA) is derived from 3-HK transamination, each molecule possessing different chemical and signaling reactivities [27,28,29]. While in mammals the accumulation of excess 3-HK is mitigated through its hydrolysis, many insects, including *D. melanogaster* and mosquitoes, lack the hydrolytic enzyme kynureninase and 3-HK can only be removed through its transamination to XA or be used in the biosynthesis of ommochrome eye pigments [30,31,32].

Tryptophan catabolism is especially relevant to adult female mosquitoes that feed on blood, a rich source of proteins, including hemoglobin. Larvae, living in an aquatic environment rich in microorganisms and proteins, also handle a significant quantity of tryptophan and store the potentially harmful 3-HK to produce ommochrome utilized in adult eye pigmentation during metamorphosis [30,32]. In *D. melanogaster*, larvae convert dietary tryptophan to KYN in the fat body, and KYN is transferred to the MTs that accumulate 3-HK [33]. These metabolites are subsequently released into the hemolymph during the pupal stage to support the production of ommochrome in the eyes and ocelli [31,34].

The well-established role of tryptophan, KYN and other derivatives in regulating immune, inflammatory, and neurological processes in humans has promoted the exploration of their autofluorescence (AF) spectral properties for the development of fast and cost-effective analytical techniques aimed at quantifying these compounds in the field of biomedicine [35,36]. Furthermore, a family of brominated derivatives of KYN, likely responsible for the skin biofluorescence of certain shark species, has been optically characterized as a foundation for addressing new questions regarding antimicrobial and photoprotective roles [37]. In insects, a light blue and intensely fluorescing material identified as KYN was initially described within the cytoplasmic “globules” found in the fat body cells of *Drosophila* pupae. The increase in AF ascribed to KYN following larval feeding on tryptophan was used as a tool to support early genetic and enzymatic studies [38,39,40]. Besides AF imaging, light spectroscopy revealed changes in the absorption spectra recorded from cell-free larval extracts. These changes were attributed to the production of KYN that depended on the larval consumption of tryptophan [38]. Subsequently, in situ AF measurements showed changes in both the amplitude and spectral shape of signals collected from MTs under a 365 nm excitation that were dependent on tryptophan, KYN or 3-HK supplementation to the larvae of eye-color mutants of *Drosophila*, supporting the importance of MTs in the storage of the ommochrome precursors in this developmental stage [41]. More recently, a study described changes in the AF material within the principal cells of the MTs of *Drosophila* larvae when observed under a 405 nm excitation, demonstrating a link between the consumption of tryptophan, the KYN pathway and, surprisingly, the intracellular storage of zinc ions [42]. Thus, the existing literature offers limited and fragmented information on the AF of compounds associated with the tryptophan–KYN pathway and the potential responses of MTs in insects fed with each of these products. Nevertheless, it is evident that UV or near-UV excitation conditions are prevalent among the spectra, with variations in emission profiles likely attributable to different specific tryptophan derivatives and to their molecular environment.

The characterization of AF signals in mosquitoes has been recently approached in anatomical structures associated with mechanical and sensory functions, with potential relevance also for vector surveillance and control [43,44,45]. A mixed and variable presence of the bluish AF ascribable to the elastic and resilient resilin and of the greenish-reddish AF ascribable to the rigid chitin has been detected in different body compartments, consistently with their already proposed ability to affect the elasticity or stiffness of different structures and their related mechanical and sensory functions [44,45,46,47,48]. The dependence of the body surface pattern on its scales, black or fluorescing with a reddish or white-bluish signal for the likely presence of melanin and its precursors or resilin, respectively, has been proposed to have implications for mosquito inter- and intra-specific communication [43,44]. Studies on the AF in the MTs of mosquitoes, on the other hand, are currently lacking but anticipated to yield insights into the metabolic and detoxification processes within these organs, shedding light on the mosquito’s physiological status and its reactions to environmental factors or treatments. Establishing connections between AF and the chemistry of responsible fluorophores is of particular interest, as the results would offer valuable models in experimental biomedicine. Interestingly, in addition to the crucial KYN transamination pathway responsible for regulating 3-HK detoxification as XA during the development of the larvae and pupae of the yellow fewer mosquito *Aedes aegypti* (Diptera: Culicidae) [32], evidence for an antioxidant role of XA during blood digestion exists in this species [49]. Female mosquitoes from different species with mutations in the KYN hydroxylase orthologs display disruptions in tryptophan catabolism and show altered microbiota, with impacts on several mosquito physiological traits [24]. The effect of blood feeding on the metal ion content was also investigated recently in *Anopheles albimanus* (Diptera: Culicidae) and *Ae. aegypti* mosquitoes [50]. Studies examining the transcriptome of MTs in adults of the Asian tiger mosquito, *Aedes albopictus* (Diptera: Culicidae), have revealed an up-regulation of the genes encoding enzymes related to tryptophan oxidation and the KYN pathway in blood-fed females. In light of the MTs’ role in excreting and detoxifying heme and nitrogen wastes from the blood, as well as the potential functions of XA as a heme and metal chelator and as an antioxidant agent, these findings carry implications for developing strategies aimed at preventing MT detoxification as a means to control disease vectors [25,51].

In the present study, the goal has been to investigate AF as a potential biomarker of the functional status of MTs during development and in response to diet. We conducted an in situ AF characterization of the MTs of *Ae. albopictus* and employed a combination of confocal imaging and microspectrofluorometry to analyze the AF signals in the MTs at different developmental stages, including larvae, pupae, sugar-fed male and female adults as well as blood-fed females. In addition, larvae fed on tryptophan have been used as models to validate the effective influence of its dietary intake and metabolism on MT AF. Our work has implications for the development of real-time physiological monitoring procedures and for the investigation of responses to environmental challenges or experimental treatments.

## 2. Results

### 2.1. Autofluorescence Imaging of the Malpighian Tubules in the Different Developmental Stages of Ae. albopictus

**Figure 1:**
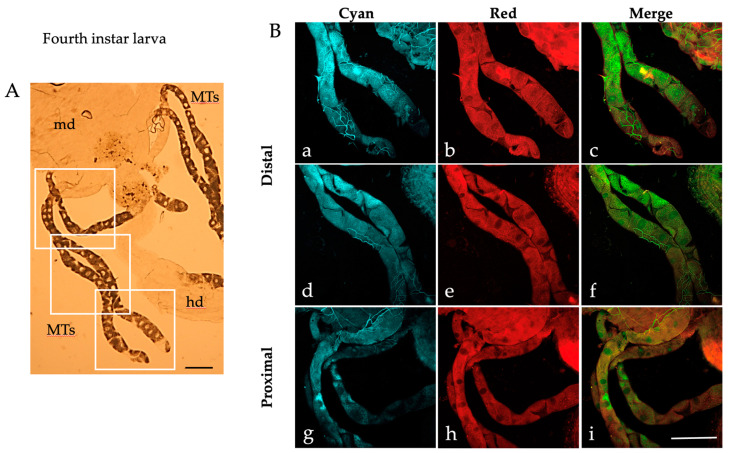
Malpighian tubules isolated from an *Ae. albopictus* fourth instar larva. (**A**) Bright field image of MTs. Frames indicate the MT areas from which AF confocal images have been collected. (**B**) AF confocal images of MTs recorded under the “Cyan” (**a**,**d**,**g**) and “Red” (**b**,**e**,**h**) conditions and respective merged images (**c**,**f**,**i**) from the distal (blind end, (**a**–**c**), middle, (**d**–**f**)) and proximal (**g**–**i**) portions. md, midgut; hd, hindgut; MTs, Malpighian tubules. Scale bar = 200 μm.

When examined under a bright field microscope, the MTs dissected from *Ae. albopictus* fourth instar larvae display a characteristic elongated structure comprising a transparent proximal segment connected to the alimentary canal and a darker distal portion culminating in a blind end (Figure 1A).

Along the MTs, it is possible to distinguish the large principal cells characterized by their dense cytoplasm surrounding a transparent nucleus, particularly noticeable in the distal portion cells (Figure 1A). The principal cells of the distal portions are also readily identified in the AF images captured by confocal microscopy in the “Cyan” condition (excitation 405 nm; emission collected between 410 and 600 nm), due to the presence of abundant brilliant granules in their cytoplasm (Figure 1B). Amid the principal cells, certain darker areas exhibit the typical “star-like” thin projection branches of the stellate cells (Figure 1B) [21]. It is also possible to appreciate the tracheae (Figure 1B), which contribute to the attachment of the MTs to the gut and whose bright bluish AF may be attributable to the presence of resilin [52]. In general, most of the principal cells within the distal portion of the MTs show highly fluorescing blue granules (Figure 1B(a,d)), while this pattern is shown by fewer cells in the proximal portion (Figure 1B(g)). Fluorescing granules are less prominent in the images obtained under the “Red” AF condition (excitation 561 nm, emission collected between 565 and 700 nm), with a prevailing distribution in the peripheral cytoplasmic region of the principal cells in both the distal (Figure 1B(b,e)) and the proximal MTs (Figure 1B(h)). Thus, different AF distribution patterns are appreciable in the merged images, where the green granules represent those that fluoresce in the “Cyan” condition, the red granules represent those that fluoresce in the “Red” condition and the orange/yellow granules represent those that fluoresce in both conditions (Figure 1B(c,f,i)).

**Figure 2:**
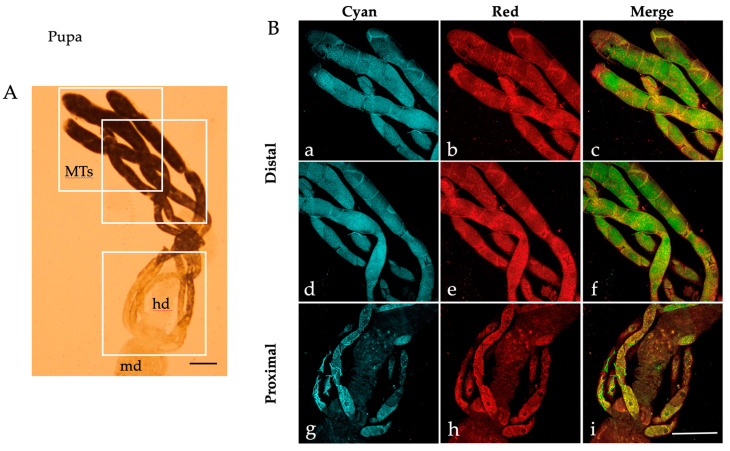
Malpighian tubules isolated from an *Ae. albopictus* pupa. (**A**) Bright field image of MTs. Frames indicate the MT areas from which AF confocal images have been collected. (**B**) AF confocal images of MTs recorded under the “Cyan” (**a**,**d**,**g**) and “Red” (**b**,**e**,**h**) conditions and respective merged images (**c**,**f**,**i**) from the distal (blind end, (**a**–**c**), middle, (**d**–**f**)) and proximal (**g**–**i**) portions. md, midgut; hd, hindgut; MTs, Malpighian tubules. Scale bar = 200 μm.

**Figure 3:**
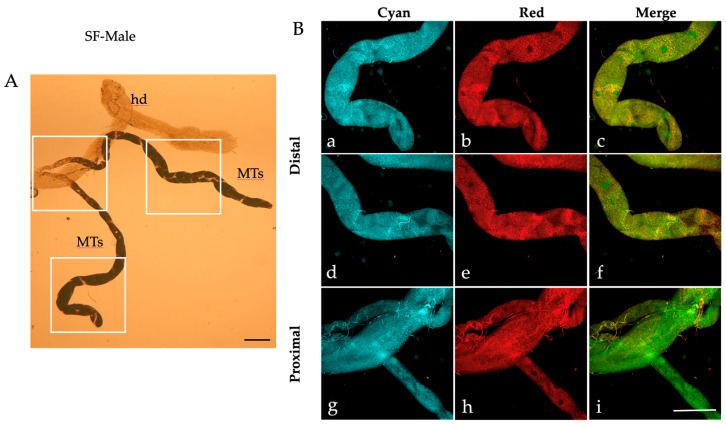
Malpighian tubules isolated from an *Ae. albopictus* adult male. (**A**) Bright field image of MTs. Frames indicate the MT areas from which AF confocal images have been collected. (**B**) AF confocal images of MTs recorded under the “Cyan” (**a**,**d**,**g**) and “Red” (**b**,**e**,**h**) conditions and respective merged images (**c**,**f**,**i**) from the distal (blind end, (**a**–**c**), middle, (**d**–**f**)) and proximal (**g**–**i**) portions. hd, hindgut; MTs, Malpighian tubules. Scale bar = 200 μm.

**Figure 4:**
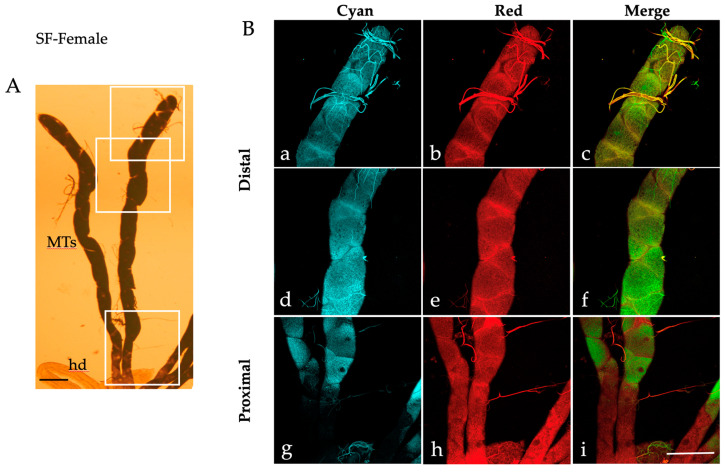
Malpighian tubules isolated from an *Ae. albopictus* sugar-fed female. (**A**) Bright field image of MTs. Frames indicate the MT areas from which AF confocal images have been collected. (**B**) AF confocal images of MTs recorded under the “Cyan” (**a**,**d**,**g**) and “Red” (**b**,**e**,**h**) conditions and respective merged images (**c**,**f**,**i**) from the distal (blind end, (**a**–**c**), middle, (**d**–**f**)) and proximal (**g**–**i**) portions. hd, hindgut; MTs, Malpighian tubules. Scale bar = 200 μm.

Similar patterns of AF granules were also observed in the MTs isolated from *Ae. albopictus* pupae (Figure 2), adult males (Figure 3) and females fed on sugar (Figure 4).

When observed under bright field conditions, MTs from the pupae and sugar-fed adults exhibit a darker and more densely packed appearance (Figure 2A, Figure 3A and Figure 4A) compared to their larval counterparts (Figure 1A). 

Moreover, AF confocal images captured from the pupae show numerous granules under the “Red” condition (Figure 2B(b,e,h)). Merge images show an extensive colocalization, particularly in the proximal portion of the pupal MTs (Figure 2B(i)), as well as in the distal portion of the sugar-fed adult MTs, more discernible in males than in females (compare Figure 3B(c,f) to Figure 4B(c,f)).

**Figure 5:**
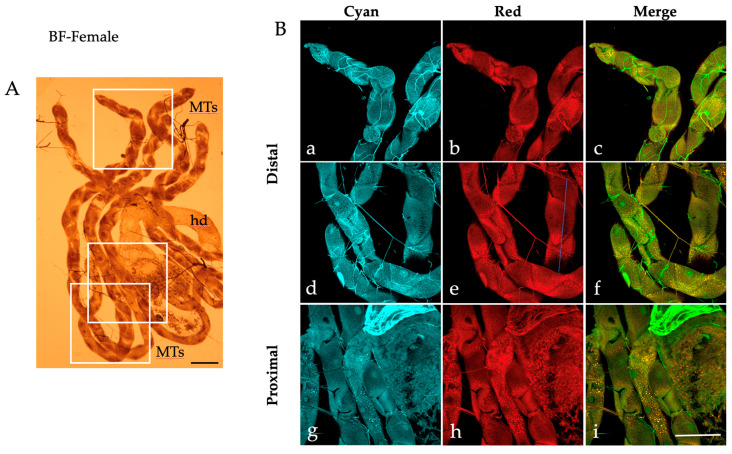
Malpighian tubules isolated from an *Ae. albopictus* blood-fed female. (**A**) Bright field image of MTs. Frames indicate the MT areas from which AF confocal images have been collected. (**B**) AF confocal images of MTs recorded under “Cyan” (**a**,**d**,**g**) and “Red” (**b**,**e**,**h**) conditions and respective merged images (**c**,**f**,**i**) from the distal (blind end, (**a**–**c**), middle, (**d**–**f**)) and proximal (**g**–**i**) portions. hd, hindgut; MTs, Malpighian tubules. Scale bar = 200 μm.

Given the essential role that MTs play, after engorging on blood, in the excretion of excess ions and water absorbed in the hemolymph and of digested nitrogen-based metabolic waste compounds [25], we also explored MTs from females two days post-blood feeding. Under bright field observation, MTs are lighter than in pupae and sugar-fed adults, facilitating the discrimination of the principal cells (Figure 5A).

Confocal AF images reveal the massive accumulation of cytoplasmic granules along the periphery of the principal cells in the MT distal portion (Figure 5B). Depending on the cell orientation, these granules appear to cluster at the edges of the darkest, star-shaped areas representing stellate cells (Figure 5B(a,b,d,e)). This pattern is noticeable in the AF images acquired under the “Red” condition and even more in the merged images (Figure 5B(c,f)). Similar patterns are also observed in the MT proximal portion, which is greatly enriched in granules compared to larvae and sugar-fed adults (Figure 5B(g–i)). We also noted that the tracheae displayed mostly a “Cyan” AF in the larval and pupal samples, while in the adults they showed both “Cyan” and “Red” AF, likely due to a variable compresence of resilin and chitin [46,48,53].

### 2.2. Microspectrofluorometry of Malpighian Tubule Autofluorescence in the Different Developmental Stages of Ae. albopictus

Autofluorescence spectra were collected from the proximal and distal portions of the MTs across *Ae. albopictus* development.

**Figure 6:**
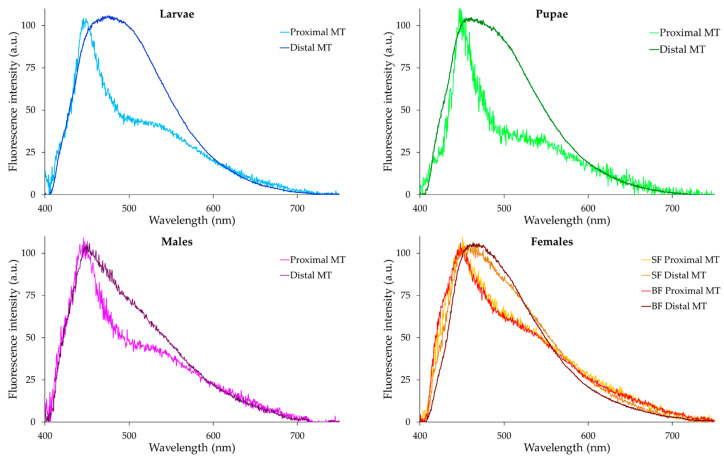
Pairs of typical AF emission spectra recorded from the proximal and distal portions of MTs dissected from the different samples of *Ae. albopictus*, as identified in each graph title. SF, sugar-fed; BF, blood-fed; MTs, Malpighian tubules. For each graph, MT proximal and distal portions are identified by colors, as shown in the legend on the right. Spectra are normalized to 100 a.u. at the maximum peak position for an easy appreciation of the relative changes in the emission profile.

The combination of pairs of spectra recorded from the two portions for each type of samples, i.e., larvae, pupae, adult males and females (both sugar- and blood-fed), allow for an appreciation of a variation in the differences between the AF emission shapes. Figure 6 shows that, in general, the AF emission of the proximal portion consists of two main bands. One band covers approximately a 410–490 nm interval, with a maximum peak around 450 nm, while the second band spans a 500–610 nm interval, with a variable amplitude between the developmental stages and sex. Conversely, the AF spectra obtained from the MT distal portion cover the overall 410–610 nm interval, likely resulting from the band peaking at about 450 nm being superimposed with additional bands at variable positions at the longer wavelengths, as suggested by the shoulders observable in the 490–610 nm region.

The spectra normalized to 100 arbitrary units (a.u.) at the values of the maximum peak position allow for better visualizing the changes in the relative contribution of the 500–610 nm band as compared to the 410–490 nm one in the AF from the proximal portion, as well as the changes in the 490–610 nm interval in the distal one.

**Figure 7:**
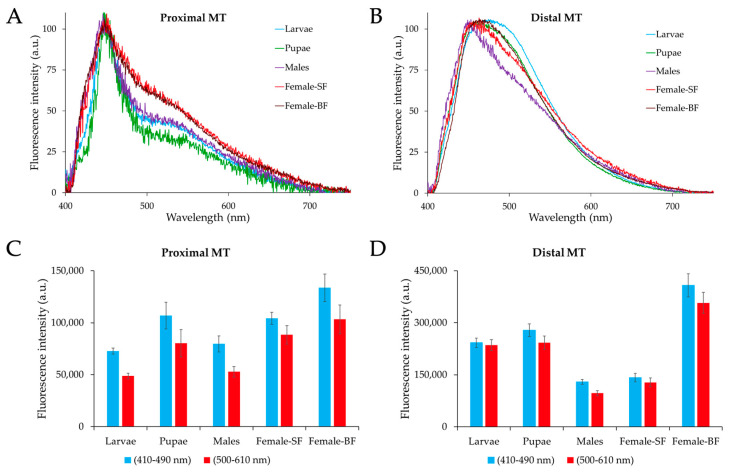
Typical examples of AF emission spectra of the proximal (**A**) or distal (**B**) portions of the MTs dissected from the different types of samples, as identified by colors, on the right. Spectra are normalized to 100 a.u. at the maximum peak position for an easy appreciation of the relative changes in the emission profile. Bar charts represent the real values (means ± S.E.) estimated from the AF emission spectra of proximal (**C**) or distal (**D**) portions of the MTs dissected from different types of samples, as identified in the x axis title. SF, sugar-fed; BF, blood-fed. Statistical analysis: significant differences (*p* < 0.05) in the case of male vs. larvae, pupae and female-BF, as well as female-SF vs. larvae, pupae and female-BF, only in the distal portion.

The presentation of all the spectra recorded from the proximal or from the distal portions in two distinct combination sets, in turn, makes it even easier to appreciate the differences between the samples (Figure 7).

As for the MT proximal portions, it can be observed that the relative amplitude of the 500–610 nm band is lowest in the pupae and highest in the female, irrespective of sugar or blood feeding (Figure 7A), while the MT distal portions exhibit pronounced variations across the different sample types in the shape of the shoulders observable in the 490–610 nm region (Figure 7B). The emission in this region varies among the different sample types; it is highest and wider in the larvae and decreases in the pupae and in sugar-fed adult females, for which blood feeding reestablishes this spectral component. The lowest amplitude in the 490–610 nm region occurs in males.

These spectral differences reflect the changes in the contribution of the various spectral components, accounting for the slight shift of the maximum peak position, from about 450 nm for the males to about 475 nm for the larvae (Figure 7B).

For the quantitative estimation of the AF signals, the values corresponding to spectral areas in the 410–490 nm and 500–610 nm intervals were integrated and are shown as bar charts (Figure 7C,D). As expected from the spectral shapes described above, area values from the 410–490 nm spectral area were generally higher than those from the 500–610 nm area for all samples, this difference being more pronounced in spectra from the proximal MTs portion, independent of the developmental stage. Proximal MT AF values varied around 100 K a.u., spectral areas being the highest in the blood-fed females and the lowest in the larvae and adult males (Figure 7C). In the MT distal portion, however, while the lowest AF values, attributable to adult males and sugar-fed females, are slightly over 100 K a.u., blood feeding results in AF values around 400 K a.u. (Figure 7D). Notably, in this region, larvae and pupae also exhibit strong AF values approaching 300 K a.u. (Figure 7D).

### 2.3. Effects of Dietary Tryptophan Administration on the AF of Larval Malpighian Tubules

Next, we investigated whether tryptophan, an amino acid previously found to be implicated in the AF of MTs in *Drosophila* [41], also affected their AF emission patterns in the Asian tiger mosquito.

**Figure 8:**
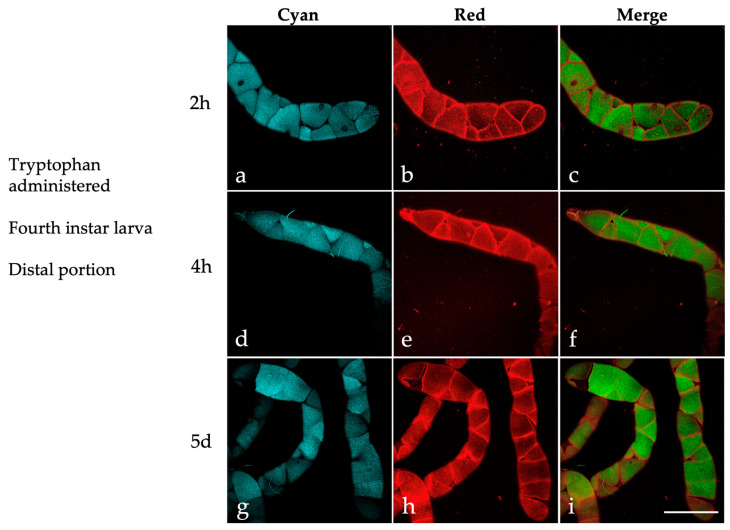
Autofluorescence confocal images of the distal portion of MTs isolated from *Ae. albopictus* fourth instar larvae administered with tryptophan (2 h, 4 h, 5-day treatment). Images recorded under the “Cyan” (**a**,**d**,**g**) and “Red” (**b**,**e**,**h**) conditions and respective merged images (**c**,**f**,**i**). Scale bar = 200 μm.

For this purpose, MTs were dissected from fourth instar larvae administered with tryptophan at 2 and 4 h post-treatment and after a 5-day exposure starting during the third instar stage. In the MT distal portion, the images acquired under the “Cyan” condition revealed well-defined dark margins that encompass the principal cells, along with their abundant content of highly fluorescent granules (Figure 8).

Remarkably, the granules are much less prominent in the “Red” compared to the “Cyan” condition, and the margins between the principal cells display strong emission in the “Red” (Figure 8b,e,h). Accordingly, in the merged images, colocalization areas are scarcely discernible (Figure 8c,f,i).

**Figure 9:**
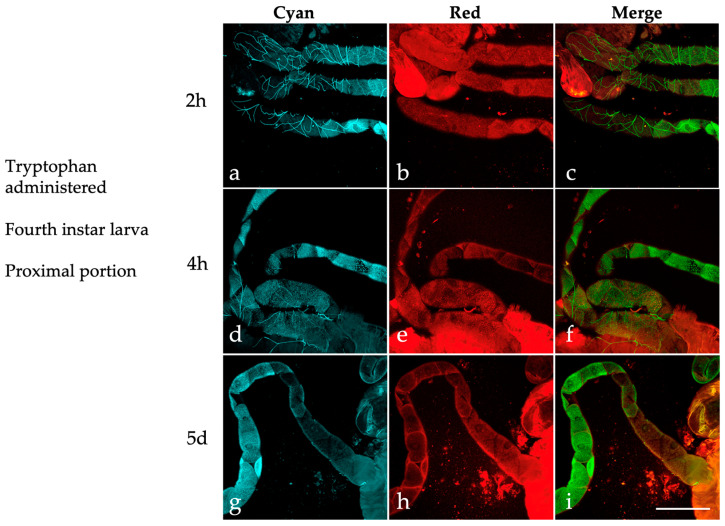
Autofluorescence confocal images of the proximal portion of MTs isolated from *Ae. albopictus* fourth instar larvae administered with tryptophan (2 h, 4 h, 5-day treatment). Images recorded under the “Cyan” (**a**,**d**,**g**) and “Red” (**b**,**e**,**h**) conditions and respective merged images (**c**,**f**,**i**). Scale bar = 200 μm.

In the proximal portion of the MTs (Figure 9), the “Cyan” condition cytoplasmic granules are, in general, less abundant than in the distal region at all three time points (Figure 9a,d,g). In some instances, the “Cyan” condition granules colocalize with those visible in the “Red” (Figure 9b,e,h), as shown in the merged images (Figure 9c,f,i).

**Figure 10:**
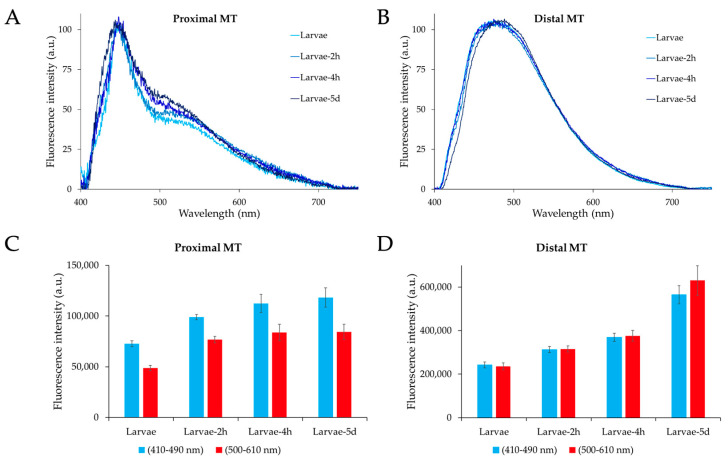
Typical examples of AF emission spectra of the proximal (**A**) or distal (**B**) portions of the MTs dissected from *Ae. albopictus* fourth instar larvae administered with tryptophan (2 h, 4 h, 5-day treatment), as identified by colors, on the right. Spectra are normalized to 100 a.u. at the maximum peak position for an easy appreciation of the relative changes in the emission profile. Bar charts represent the real values (means ± S.E.) estimated from the AF emission spectra of proximal (**C**) or distal (**D**) portions of the MTs dissected from different type of samples, as identified in the x axis title. Statistical analysis: significant differences (*p* < 0.05) in the case of larvae reared under standard conditions vs. tryptophan-administered larvae, at all time points, only in the MT distal portion; 5-day tryptophan-administered larvae vs. all the other samples, only in the MT distal portion.

The profiles of the AF emission recorded by microspectrofluorometry from the MTs of tryptophan-fed larvae were generally similar to those obtained from the larvae reared at standard conditions (Figure 10).

It should be noted, however, that the spectra from the MT proximal portions show a continuous increase in the relative amplitude values in the 500–610 nm region as compared with those in the 410–490 nm region, in a direct dependence on the time of tryptophan administration (Figure 10A). Conversely, no remarkable spectral shape changes occur in the distal portion between the larvae reared at standard conditions and those following tryptophan administration. As compared to the other samples, only the MTs isolated after 5 d of tryptophan administration show a slight narrowing at the short-wavelength side of the spectrum, with a red shift in the peak maximum value from the roughly 475 nm of the other samples to about 490 nm (Figure 10B).

The most notable effects of tryptophan administration consisted in the remarkable increase in the emission signal in a direct relationship with the time of measurement (Figure 10C,D). Of note, the increase in the AF amplitudes was much higher in the distal than in the proximal portion (Figure 10C,D), suggesting a different accumulation of AF material following dietary tryptophan administration.

## 3. Discussion

The present study represents the first attempt to describe the AF properties of the MTs of the Asian tiger mosquito, *Ae. albopictus,* in different developmental stages and after nutritional manipulations. Collectively, our results advance knowledge of the AF in these organs, which has been previously studied in other insect species [42,54,55,56] but was unexplored in mosquitoes. Our results provide the background for experiments aimed at identifying and characterizing the fluorophores responsible for the reported AF and a platform for developing and testing novel hypotheses about the function of the MTs in this key arboviral vector.

The AF confocal images of the MTs of all *Ae. albopictus* developmental stages exhibit prominent, brightly fluorescing granules, which are henceforth referred to as either “Cyan” or “Red” granules depending on the AF observation conditions. Our data show, in certain instances, the colocalization of “Cyan” and “Red” signals in the same granules. As a first comment, the intracellular localization of fluorescing granules enables us to differentiate between principal cells and darker areas associated with stellate cells. This finding may be in accordance with the role ascribed to these cells in insects, such as *Drosophila*, where stellate cells have been found to be a specialized route for rapid water flux via aquaporin channels and not a storage site [57,58]. Similarly, in mosquitoes, the principal cells, in general, contribute to the active transport of K^+^ and Na^+^ ions from the hemolymph to the MT lumen, creating an electrochemical gradient for anion transport (mostly Cl^−^) through the stellate cells [25,59], which are mitochondrion-poor and extend four projections intercalating between the principal cells.

In general, “Cyan” granules are prevalent in the principal cells of the distal portions of the MTs as opposed to the proximal ones, with some variability depending on the mosquito development and nutritional state. This observation is consistent with the functional involvement of the proximal portion, which is responsible for water and electrolyte absorption from the hemolymph as well as for the excretion of metabolites and catabolites into the MT lumen. Of note, among the excreted compounds, bluish fluorescent derivatives of tryptophan and KYN have been previously reported in the case of the MTs of *Drosophila* [20,21,41].

In *Ae. albopictus* larvae, the proximal portion of the MTs exhibits diverse and sometimes contrasting AF patterns in the principal cells, which may either have a sparse presence or a high concentration of “Cyan” and “Red” granules, with little colocalization. In contrast, the merge images from the proximal portion of pupae and blood-fed females often reveal the simultaneous presence of blue and orange-red AF material in the same granules. These “double AF” granules are also evident in some cells within the distal portions of the MTs. In sugar-fed adult mosquitoes, “Cyan” granules are less common in the proximal compared to the distal portion.

Further investigations are required to characterize and identify the specific fluorophores responsible for the various AF emission properties observed. Emissions ranging from yellow to orange-red may originate from lipofuscins and lipofuscin-like lipopigments [60] that could arise from digestive or autophagy processes and related catabolic processes during metamorphosis or after blood feeding [61,62]. In this respect, blood or non-blood feeding behaviors can affect the acquisition of pathogens differently, with important consequences on their transmission. Conversely, mutual benefits can derive from microbiota helping in digestion and diet supplementation, in exchange for a food source and protection against the mosquito gut immune response [63,64,65]. In addition, immunity is known to be modulated by a mechanism based on a crosslinked mucin layer catalyzed by the anopheline typical heme peroxidases HPX15, as assessed in the malaria vectors *Anopheles gambiae* and *An. stephensi* [66,67]. In *An. stephensi*, in particular, the modulation of AsHPX15 expression in the midgut depending on blood feeding and microbial supplementation as compared to sugar-fed females has led to the proposal of the manipulation of such immune-related pathways as an investigative approach for the control of *Anopheles* mosquitoes and their vectorial capacity [67]. The modulation of these interlaced pathways, in turn, are likely impacting the MT role in excreting catabolic wastes and related AF properties. In this context, and given a recent report on the strict relationship between environmental microbiota and their specific distribution in the MTs and midgut of *Ae. albopictus* [68], AF analysis of the MTs can be expected as a potential valuable support for investigating changes in the metabolic engagement of mosquitoes.

The unique metabolic functions of the MTs in relation to tryptophan suggest that the fluorescent products associated with this amino acid contribute to the AF of the principal cell granules. Previous research on the AF of *Drosophila* eye color mutants, as well as the in-solution characterization of ommochromes extracted from insects, have revealed AF spectra that span from the blue to approximately 550 nm [41,69]. Therefore, the variations in the presence of “Cyan” and “Red” granules, as well as their colocalization among the developmental stages we considered, could be, at least in part, attributed to metabolic and catabolic processes involving tryptophan and its derivatives. This suggestion is supported by the AF images captured in the MTs of larvae administered with tryptophan at subsequent time points, resulting in a marked increase in “Cyan” and “Red” granules in the distal portion of the MTs. The non-overlapping spatial distribution of these two types of granules, following the addition of tryptophan to the diet, is another striking observation of this study that requires further investigation. Of note, dietary KYN, a major tryptophan derivative, was directly implicated in the formation of AF “Cyan” granules in the MTs of *Drosophila* [42], and just like the case of our mosquito MTs, the *Drosophila* organs also demonstrate regional variability in their AF patterns [70].

While AF patterns provide a qualitative understanding of the distribution of “Cyan” and “Red” granules, the images pose challenges for a quantitative analysis of the AF signal. This is due to several factors, including the granules’ three-dimensional distribution across multiple focal planes, their high spatial density in many MT regions and the need to fine-tune excitation energy for satisfactory image capture. As a result, estimating the AF signal in terms of the overall emission or even in terms of the occupied areas in a single plane is problematic. To overcome this challenge, we have employed microspectrofluorimetric analysis, which not only allows for the estimation of their quantitative contributions to the overall AF emission but also provides insights into the emission profile and the underlying spectral components.

Microspectrofluorometric results support the contribution of tryptophan metabolites to the AF of the MTs. The AF spectra obtained from the proximal portions of the MTs typically exhibit a broad profile, covering the 410–610 nm spectral range, with a sharp band peaking around 450 nm and a well-defined shoulder in the 500–610 nm interval. In contrast, AF spectra from the distal portions encompass the overall 410–610 nm interval, with a variable contribution from the 500–610 nm component depending on the type of sample under consideration. These results are consistent with the few examples of AF spectra reported in the literature for tryptophan and its metabolites as pure compounds, which anyway show some variability in the peak position and shape that can be ascribed to the effects of solvents as compared to the molecular micro-environmental conditions in granules [35,36]. Also, the literature reports on the AF microspectrofluorometric in situ characterization of the MTs of *Drosophila*, showing wide spectra in the 430–550 nm interval or a narrower band peaking just below 450 nm in the wild-type Oregon-R strain and in the *vermillion brown* eye-color mutants, respectively. Notably, the *vermilion brown* eye-color mutant showed a widening and a red shift of the 450 nm peaking band after the larvae were fed with KYN or 3-HK in the presence of tryptophan, respectively [41]. Spectra taken directly from individual granules under similar conditions showed a peak around 510 nm [42]. In our study, the involvement of tryptophan-derived metabolites in the AF of the MTs is consistent with the changes in the contribution of the 500–610 nm component to the overall spectral emission obtained from the MT distal portions across development. Indeed, the lowest contribution of the 500–610 nm component detected in sugar-fed adults, according to both the normalized spectra and the absolute AF values, can be attributed to their reduced metabolic activity compared to the active engagement of larvae and blood-fed females processing protein-rich food from microorganisms and organic detritus in the aquatic environment or blood, respectively [71].

The differences in both the AF images and spectral results between the proximal and distal portions are consistent with the known anatomical separation of MT physiological functions [20,21]. The likely relationship between AF and the MT prevailing functional roles in providing for osmoregulation, or for the excretion of wastes and xenobiotics, is supported by the emission being significantly higher and more dynamic in the hemolymph-absorbing distal portion following larval tryptophan treatment or female blood feeding.

## 4. Materials and Methods

### 4.1. Insects and Malpighian Tubule Sample Preparation

The *Ae. albopictus* RER strain [72], obtained from the Centro Agricoltura Ambiente (CAA) “G. Nicoli” (Crevalcore, Italy), was used in this study. Larvae were reared with fish food pellets (Goldfish Granules, Tetra GmbH, Melle, Germany). Emerging adults were reared following standard protocols (see [73]), i.e., at a temperature of 26 ± 1 °C, 70% relative humidity and a photoperiod of 12:12 h light:dark, and maintained on a 20% sugar solution. Females were blood-fed with defibrinated mutton blood (Biolife Italiana S.r.l., Milan, Italy) and a membrane feeding system.

Individual fourth instar larvae (*n* = 20), pupae (*n* = 20) and 4–6-day-old adults (*n* = 10 sugar-fed males; *n* = 10 sugar-fed females; *n* = 10 blood-fed females) were cold-anesthetized to proceed to the dissection of all five MTs using fine forceps (Dumont #5SF, Fine Science Tools, Foster City, CA, USA) under a stereomicroscope (Olympus SZ40, Olympus Optical Co. GmbH, Hamburg, Germany). Each individual was transferred on a glass slide with a drop of 1X phosphate buffer saline (PBS) solution. The rectum was gently pulled to release from the gut and the MTs the abdominal cavity. The MTs were then separated from the tracheae, fat body and muscles. Particular attention was applied in removing the tracheae, except those coiled around the MTs, which were not removed to limit damage to these organs. The MTs were dissected from engorged females 2 days after blood feeding.

In a parallel experiment, MTs were dissected from tryptophan-fed fourth instar larvae. Fifty larvae were reared in water supplemented with L-tryptophan (Sigma Aldrich, Milan, Italy) at a final concentration of 2.5 mM and dissected 2 (*n* = 10 larvae) and 4 (*n* = 10) hours after the tryptophan addition. An additional batch of third instar larvae was reared in water supplemented with tryptophan at the abovementioned concentration and dissected as fourth instars (*n* = 10 larvae) to isolate the MTs during the same stage but following five days of dietary treatment.

Once isolated, the MTs were immediately subjected to a mild fixation by transferring all five MTs in a drop of 4% formaldehyde (Sigma Aldrich, Milan, Italy) in PBS solution [42]. This procedure lasted 10 min, and it was chosen as the best compromise to preserve both the MT structure and AF properties, based on the literature and of a set of preliminary experiments comparing AF from unfixed and fixed samples. For AF analysis, the samples were then mounted on a glass slide, and the coverslip was sealed with nail polish.

### 4.2. Bright Field and AF Confocal Microscopy Imaging

Bright field images were collected from MTs by means of an Olympus fluorescence microscope (BX53 model, Olympus Optical Co. GmbH, Hamburg, Germany) with the Olympus objective Plan 4X (numerical aperture, n.a., 0.10). Autofluorescence images were acquired using a Zeiss confocal microscope (LSM800 model, Zen Blue 2.6 software, Carl Zeiss S.p.A., Milan, Italy) with a Zeiss Plan-Apochromat 20X objective (n.a. 0.8), according to the Z staking modality. The AF acquisition conditions were an excitation of 405 nm and emission of 410–600 nm (“Cyan” condition) and an excitation of 561 nm and emission of 565–700 nm (“Red” condition). The AF acquisitions in the two conditions were taken subsequently from the same field. The images were stored in a portable memory drive and then transferred to a PC for their processing by means of the FiJi/ImageJ software (version 1.53t; [74]). The images collected under the “Cyan” and “Red” conditions are shown in false colors, while merged images have been obtained after converting “Cyan” images into false green, to obtain possible overlapping areas in the easily appreciable yellow. For presentation, the images were assembled in panels using Power Point (Microsoft^®^, version 16.78).

### 4.3. Spectrofluorimetric Analysis

The AF emission spectra were obtained from MT samples dissected from fourth instar larvae (*n* = 10), pupae (*n* = 10), adult males (*n* = 5) and adult females (*n* = 5 for sugar-fed (SF) and *n* = 5 for blood-fed (BF) individuals), respectively. The AF signals were measured by means of a multichannel analyzer (Hamamatzu PMA-12 photonic model, Hamamatsu Photonics Italia Srl, Arese, Italy), operating in epiillumination with a Leica orthoplan fluorescence microscope (Leica Microsystems S.r.l., Buccinasco, MI, Italy) and a Leica objective (40×, n.a. 0.65). The 366 nm excitation was provided by an LC-L1 UV-LED light source, coupled with an LED head unit cable guided to the excitation entrance of the Leica fluorescence microscope. The AF signals were recorded in the 410–750 nm visible light interval range from the MTs (0.008 × 10^−3^ mm^2^ diaphragmed area) through a 50/50 dichroic mirror and a 405 nm barrier filter. Data were stored on a portable memory drive and then processed by means of the Excel program (Microsoft^®^, version 16.78). The values of the emission signals of the selected spectral regions were calculated from the original spectra in terms of integrated area values (means ± standard error (S.E.)). Statistical analysis was performed by means of the Kruskal–Wallis One-Way ANOVA test, with Dunn’s test as a post hoc test for multiple-comparison to denote significantly different pairs (Unistat^®^ Statistical Package, version 5.0.15, Unistat Ltd, London, UK); the value of *p* < 0.05 was considered to indicate statistical significance. For the presentation, the spectra were normalized to 100 arbitrary units (a.u.) at their maximum peak to allow for an easy appreciation of changes in the shape of the profiles.

## 5. Conclusions

Our investigation into the AF properties of the MTs of *Ae. albopictus* has revealed distinctive highly fluorescing granules with different patterns of distribution across development and in response to dietary manipulations, as well as between proximal and distal portions of the MTs for each sample type. Here, AF spectroscopy provided further insights indicating distinct spectral profiles with an AF band peaking at about 450 nm, combined with a 500–610 nm component of a variable amplitude. This spectral evidence is consistent with the few literature reports on the effects of the administration of tryptophan or its derivatives on the AF of the organs engaged in their metabolism. Further support of the relationship between tryptophan and its derivatives and MT AF is provided by the impact of tryptophan administration to larvae on the AF patterns of their MTs, accompanied by an increase in the AF amplitude, especially in the distal MT portion. The spatial separation of “Cyan” and “Red” granules shown by confocal imaging, in turn, supports a dynamic response to tryptophan in relation to metabolic processes. This pioneering effort in a systematic characterization of the AF of MTs in *Ae. albopictus* during development and after nutritional manipulation provides a basis for future investigations into the specific tryptophan derivatives acting as fluorophores responsible for the distinctive AF patterns. The identification of these fluorophores could contribute to further understanding the physiological functions of the MTs, with particular reference to the impact of dietary factors and the management of nitrogen wastes and metals, as well as the related antioxidant and detoxification roles in this crucial arboviral vector. Unravelling the mechanisms underlying variations in the AF of MTs in response to environmental factors or treatments and their relationship with specific metabolic alterations may inspire novel applications of AF markers in insect biology and biomedicine.

## Data Availability

The data presented in this study are available in the article. The raw spectral data are available upon request.
